# Integrated transcriptomic and metabolomic profiling of *Breynia androgyna* (L.) tissues

**DOI:** 10.1186/s13104-025-07521-8

**Published:** 2025-10-27

**Authors:** Darvien Gunasekaran, Syazwani Basir, Noraini Talip, Ahmad Bazli Ramzi, Syarul Nataqain Baharum, Hamidun Bunawan

**Affiliations:** 1 School of Biological Sciences, Faculty of Applied Sciences, Universiti Teknologi MARA (UiTM), Cawangan Negeri Sembilan, Kampus Kuala Pilah, Kuala Pilah, Negeri Sembilan 72000 Malaysia; 2 Department of Biological Sciences and Biotechnology, Universiti Kebangsaan Malaysia, Bangi, 43600 UKM Selangor Malaysia; 3 Institute of Systems Biology, Universiti Kebangsaan Malaysia, Bangi, 43600 UKM Selangor Malaysia

**Keywords:** Breynia androgyna, Transcriptomic, LC-MS metabolomics, Secondary metabolites

## Abstract

**Objectives:**

*Breynia androgyna* (L.) is a perennial shrub renowned for its ethnomedicinal properties and nutritional value. Despite its importance, genomic and metabolomic information for this species remains limited. This study presents newly generated transcriptomic and metabolomic datasets derived from multiple tissue types to facilitate functional annotation, gene discovery, and elucidation of secondary metabolite biosynthesis pathways.

**Data description:**

For transcriptomics analysis, total RNA was extracted from leaf, flower, fruit, and stem tissues of *B. androgyna*, followed by high-throughput sequencing using the Illumina HiSeq platform. De novo transcriptome assembly and functional annotation of the obtained reads yielded a high-quality unigene dataset with comprehensive coverage of key metabolic and regulatory pathways. Gene Ontology (GO) and KEGG pathway analysis indicated active involvement in both primary and secondary metabolic processes. In parallel, liquid chromatography-mass spectrometry (LC-MS) profiling was performed for each tissue type, providing complementary chemical characterization and metabolite insights. These integrated datasets deliver a multilayered molecular perspective of *B. androgyna*, supporting future research into its biosynthetic pathways. All raw RNA-seq data are publicly available in the NCBI Sequence Read Archive (SRA) under BioProject accession PRJNA494978. The LC-MS spectral data are accessible in csv. and Excel file format. The integration of transcriptomic and metabolomic profiling provides a comprehensive molecular resource for *B. androgyna*. This multidimensional dataset facilitates the investigation of gene function, regulatory mechanisms of metabolic pathways, and the molecular basis underlying its ethnomedicinal properties.

## Objective

*Breynia androgyna* (L.) Merr., commonly known as star gooseberry or katuk, is a perennial shrub native to Southeast Asia that has gained considerable attention for its nutritional and ethnomedicinal properties. Traditionally consumed as a leafy vegetable, *B. androgyna* is characterised by its rich content of bioactive compounds, including flavonoids and phenolic acids, which are associated with potent antioxidant, anti-inflammatory, and antimicrobial activities [1]. Ethnomedically, it has been employed to promote lactation, bolster immune responses, and manage metabolic disorders [2]. Despite its widespread utilization and pharmacological potential, the molecular mechanisms for the biosynthesis of its key phytochemicals remain largely unexplored, underscoring the need for comprehensive molecular characterization to elucidate its biosynthetic pathways and functional genomics.

With the emergence of high-throughput sequencing and mass spectrometry technologies, it is now feasible to generate comprehensive multi-omics datasets for non-model species. Transcriptomic analysis, particularly through de novo assembly using RNA sequencing (RNA-Seq), facilitates the identification of coding sequences, gene families, and biosynthetic pathway components in species lacking a reference genome [3]. Simultaneously, liquid chromatography–mass spectrometry (LC–MS)-based metabolomics enables the untargeted profiling of small molecules, offering insights into the metabolic diversity and complexity of plant tissues [4, 5].

In this study, we provide a dataset comprising a de novo assembled transcriptome and untargeted LC–MS metabolomic profiles from different tissues of *B. androgyna*. The integration of these datasets offers a foundational resource for elucidating the molecular and metabolic networks associated with the plant’s bioactivity. These data are intended to support future research in plant functional genomics, phytochemistry, and systems biology, particularly in the context of medicinal and underutilized plant species.

## Data description

### Plant cultivation and sample collection

*Breynia androgyna* cuttings were cultivated and established in open-field plots under ambient outdoor conditions at Universiti Kebangsaan Malaysia (UKM), Bangi, Malaysia. Tissue samples, including leaves, stems, flowers, and fruits were harvested at the flowering stage, approximately ten weeks post-planting. Immediately following collection, samples were processed to preserve RNA integrity and metabolite stability for subsequent metabolomic and transcriptomic analyses.

### Extraction of secondary metabolites

For metabolite profiling, each of the four tissue types (leaf, stem, flower, and fruit) was independently ground into a fine powder and subjected to extraction with acidified methanol, following a modified protocol adapted from [6]. The extracts were vortexed and subsequently sonicated using an ultrasonic cleaner (Model S30H, Elma, Singen, Germany) to enhance metabolite solubilization. After sonication, samples were centrifuged, and the supernatants were filtered and transferred into LC-MS autosampler vials. Each tissue sample was prepared in triplicate biological replicates to ensure analytical reproducibility. All solvents and reagents employed in extraction and analysis were of high-performance liquid chromatography (HPLC) grade.

### LC–MS instrumentation and data processing

Chromatographic separation was performed using a Thermo Scientific Acclaim™ Polar Advantage II C18 column (3 × 150 mm, 3 μm particle size) using a Dionex UltiMate 3000 UPLC system (Thermo Fisher Scientific, USA). Mass spectrometric detection was conducted using a MicrOTOF-Q III instrument (Bruker Daltonik GmbH, Germany) operated in positive electrospray ionization (ESI+) mode. Mass spectra were acquired over an m/z range of 50–1000. Raw data files were processed with Compass Data Analysis software version 4.2 (Bruker, Germany), following the protocol described by [7]. The processed data were converted to comma-separated values (CSV) format for downstream analysis. Data preprocessing involved applying a signal-to-noise (S/N) ratio threshold of 10 and a smoothing width of 3 across the entire dataset. Normalization was performed using vanillic acid at a concentration of 100 mg/mL as the internal standard, which was a reference feature common to all samples. The normalized data were then subjected to statistical analysis, including one-way analysis of variance (ANOVA) and multivariate analysis. Prior to principal component analysis (PCA), Pareto scaling was applied to the normalized data matrix to mitigate the influence of large intensity differences among metabolites. All statistical analyses were conducted using the MetaboAnalyst version 3.0 platform [8]. The LC-MS profiling identified 682 metabolite peaks across *B. androgyna* tissue extracts, of which 233 peaks exhibited statistically significant differences (*p* < 0.05). PCA revealed distinct clustering according to tissue type, with the first two principal components (PC1 and PC2) accounting for over 80% of the total variance. Notably, fruit and flower tissues showed overlapping clusters, indicating a high degree of biochemical similarity.

### RNA extraction and quality assessment

Total RNA was isolated from leaf, stem, flower, and fruit tissues of *B. androgyna* using a modified protocol adapted from [9]. The integrity of the extracted RNA was initially assessed via 0.1% agarose gel electrophoresis. Subsequently, RNA integrity was evaluated using an Agilent 2100 Bioanalyzer (Agilent Technologies, CA, USA). Samples exhibiting an RNA Integrity Number (RIN) greater than 7.0 and 28 S/18S ratios exceeding 1.8 were considered suitable for next-generation sequencing [10]. RNA quantification and purity were further confirmed by Nanodrop spectrophotometry, with all samples demonstrating concentrations exceeding 30 ng/ul and acceptable absorbance ratios within the range of 1.8–2.2 for 260/280 and 260/230 ratios. Only RNA samples from *B. androgyna* meeting these stringent quality criteria were selected for downstream transcriptome sequencing.

### cDNA library construction, sequencing and de Novo assembly

Strand-specific cDNA libraries were constructed using the SureSelect RNA Library Preparation Kit (Agilent Technologies, CA, USA). Messenger RNA (mRNA) was enriched using oligo(dT) magnetic beads and subsequently subjected to enzymatic fragmentation. Adapter sequences were ligated to both termini of the synthesized cDNA fragments using SureSelect oligo adaptors. The prepared libraries were sequenced on the Illumina HiSeq 4000 platform (paired-end, 150 bp reads) by Theragene Etex (Seoul, Korea). Raw sequencing reads were trimmed to remove adapter sequences using Cutadapt and reads with a Phred quality score below Q20 were discarded to ensure data quality. High-quality reads were de novo assembled using Trinity version 2.0.2 [3]. Redundant transcripts were clustered, and the longest isoform within each cluster was selected as the representative unigene.

For *B. androgyna*, sequencing generated 260,432,434 raw reads, which after quality filtering, resulted in 233,144,516 high-confidence clean reads. Post-assembly, unigenes were analysed for length distribution and coding potential. The majority of unigenes ranged from 200 to 500 bp in length. Coding sequences (CDS) were predicted using TransDecoder, facilitating downstream functional annotation and pathway analysis.

### Functional annotation

Functional annotation of the assembled unigenes was performed through sequence similarity searches against major public protein databases, including NCBI non-redundant (Nr), Swiss-Prot, InterPro, KEGG, and Gene Ontology (GO). BLASTx analysis was conducted with an e-value cutoff of 1e − 5 to ensure high-confidence matches. Approximately 72.5% of *B. androgyna* unigenes exhibited significant homology with entries in the Nr database, while 50.2% matched sequences in Swiss-Prot. Additional functional annotations using InterPro, KEGG, and GO databases identified annotations for 39.1%, 29.6%, and 44.8% of unigenes, respectively, providing insights into potential gene functions, biological pathways, and molecular processes.

The results of sequencing and pre-processing alongside metabolomics data are summarized in Table 1 below.

**Table 1:** Overview of data files/data sets

Label	Name of data file/data set	File types (file extension)	Data repository and identifier (DOI or accession number)
Data set 1	Rna seq SA_leaf	FASTQ (.fastq)	http://identifiers.org/insdc.sra: SRX4814390
Data set 2	Rna seq SA_fig	FASTQ (.fastq)	http://identifiers.org/insdc.sra: SRX4814391
Data set 3	Rna seq SA_flower	FASTQ (.fastq)	http://identifiers.org/insdc.sra: SRX4814392
Data set 4	Rna seq SA_stem	FASTQ (.fastq)	http://identifiers.org/insdc.sra: SRX4814393
Data file 1	*Breynia androgyna* LC-MS data	Excel file (.csv)	10.6084/m9.figshare.29595299 [11]
Data file 2	PCA score plot of *Breynia androgyna* tissues	Image file (.png)	10.6084/m9.figshare.29595299 [11]
Data file 3	Nanodrop and Bioanalyzer Readings for RNA Extracts from Different Tissues *Breynia androgyna*	Document file (.docx)	10.6084/m9.figshare.29595299 [11]
Data file 4	Quality Control and Filtering of High-Quality Reads from Transcriptome Sequencing	Document file (.docx)	10.6084/m9.figshare.29595299 [11]
Data file 5	Summary of Transcriptome Assembly Statistics for *Breynia androgyna*	Document file (.docx)	10.6084/m9.figshare.29595299 [11]
Data file 6	Annotation Statistics Using Protein Databases of *Breynia androgyna*	Document file (.docx)	10.6084/m9.figshare.29595299 [11]
Data file 7	Unigenes transcript of *Breynia androgyna*	Excel file (.csv)	10.6084/m9.figshare.29595299 [11]

### Limitations

The transcriptomic dataset presented in this study provides foundational insight into gene expression profiles across four tissues of *Breynia androgyna* - namely leaf, flower, fruit, and stem. However, several limitations should be acknowledged:

1. Tissue-Specific and Developmental Stage Limitations: RNA samples were collected from these tissues at a single developmental stage (flowering), which constrains the ability to capture temporal; or environmental variations in gene expression and limits insights into dynamic transcriptomics changes over developmental stages or under different environmental conditions.
2. Absence of Biological Replicates: The dataset lacks biological replicates, which diminishes its immediate utility for robust differential expression analysis. Nevertheless, the high sequencing depth ensures reliable gene identification and comprehensive transcriptome assembly. The quality of transcript annotation, combined with stringent quality control metrics, enhances the dataset’s reliability and interpretability. Despite the absence of replication, this resource serves as a valuable reference for future studies on tissue-specific gene expression, secondary metabolite biosynthesis, and comparative genomics in *B. androgyna*. Researchers are encouraged to utilise this dataset as a baseline for designing experiments incorporating biological replicates, validation assays, and extended functional analyses.
3. Annotation Coverage Constraints: Functional annotation was performed using public databases such as Swiss-Prot and NCBI NR. However, the limited genomic resources for *B. androgyna* and closely related taxa may restrict annotation completeness, particularly for species-specific or novel transcripts.
4. Lack of Experimental Validation: The expression levels and functional predictions of identified genes have not been validated through independent experimental techniques such as quantitative PCR or in situ hybridization, underscoring the need for further validation in future research.

Despite these limitations, the raw sequencing data (accessible via NCBI SRA under accession numbers SRX4814390–SRX4814393 under BioProject PRJNA494978) constitute a valuable resource for advancing genomic, functional, and evolutionary research in *B. androgyna* and related species within Euphorbiaceae family.

## Data Availability

The raw fastq files were deposited in the National Center for Biotechnology Information and are available with accession numbers of SRX4814390 – SRX4814393 for leaf, fruit, flower and stem, respectively, under Bioproject PRJNA494978.

## Abbreviations

SRA: Short read archive
KEGG: Kyoto Encyclopedia of Genes and Genomes
GO: Gene Ontology
CDS: Coding sequences
csv.: Comma-separated values
